# ZKSCAN3 promotes ovarian cancer cell proliferation by increasing HSPB1 expression

**DOI:** 10.3389/fmolb.2025.1623062

**Published:** 2025-11-28

**Authors:** Qian Ke, Zhenyong Li, Li Fan, Neng Li, Lidong Sun, Hongbo Zhao, Tanjing Song

**Affiliations:** 1 Department of Biochemistry and Molecular Biology, School of Basic Medicine, Tongji Medical College, Huazhong University of Science and Technology, Wuhan, China; 2 Wuhan Children’s Hospital, Tongji Medical College, Huazhong University of Science and Technology, Wuhan, China; 3 Cell Architecture Research Institute, Huazhong University of Science and Technology, Wuhan, China; 4 Obstetrics & Gynecology Hospital of Fudan University, Shanghai Key Lab of Reproduction and Development, Shanghai Key Lab of Female Reproductive Endocrine Related Diseases, Shanghai, China

**Keywords:** ZKSCAN3, transcription factor, ovarian cancer, HSPB1, cell proliferation

## Abstract

Ovarian cancer has the worst prognosis among major gynecological cancers. Current therapies include platinum, Taxol, angiogenesis inhibitors, and poly[ADP-ribose]polymerase (PARP) inhibitors. However, resistance develops in most ovarian cancer patients. Identification of more pro-tumor factors in ovarian cancer may provide insights into ovarian cancer biology and therapy. In this study, we find ZKSCAN3, a zinc-finger transcription factor, is overexpressed in ovarian cancer. We show that ZKSCAN3 promotes ovarian cancer cell proliferation. Through RNA-Seq and chromatin immunoprecipitation (ChIP)-seq, HSPB1 is identified as a target gene of ZKSCAN3. HSPB1 expression is significantly decreased upon suppressing ZKSCAN3 expression. Suppressing HSPB1 expression also inhibits ovarian cancer cell proliferation. In contrast, expressing exogenous HSPB1 partially rescues the cell proliferation in ZKSCAN3 knockdown cells, which supports HSPB1 as a functional target gene of ZKSCAN3. Collectively, our study uncovers a functional ZKSCAN3-HSPB1 axis that promotes ovarian cancer cell proliferation.

## 1 Introduction

Ovarian cancer is one of the major types of gynecological cancers (Chen et al., 2016; Sung et al., 2021). It has a higher mortality rate than cervical and endometrial cancers despite a lower occurrence rate (Chen et al., 2016; Sung et al., 2021). Currently, there is no practical early screening for ovarian cancer. As a result, most patients are diagnosed at a late stage of the disease (Gourley and Bookman, 2019). In the past, there were only limited options for ovarian cancer pharmaceutical therapy. Platinum, Taxol, and angiogenesis inhibitors have been the mainstay (Gourley and Bookman, 2019). However, resistance exists or develops in most patients. Recent adoption of poly [ADP-ribose] polymerase (PARP) inhibitors and immunotherapy faces challenges of non-response and/or side effects (George et al., 2017; Gourley and Bookman, 2019). Collectively, there is a huge unmet medical need for ovarian cancer patients. Further insights into ovarian cancer biology may lead to new therapeutic strategies. Hotspot mutations in classical oncogenes, such as NRAS, and tumor suppressor genes, such as RB1 and TP53, are observed in some ovarian cancers (Zhang et al., 2016). However, the mutation rates vary and do not explain most of the sporadic diseases. Transcription factors, which represent over 10% of protein-coding genes in the human genome, regulate gene expression to affect cancer progression. In the past, dysregulation of transcription factors such as FOXM1 and MYC has been linked to ovarian cancer initiation and progression (Nameki et al., 2021). Yet, the function of the majority of transcription factors in ovarian cancer remains unknown.

ZKSCAN3 is a transcription factor that contains a zinc-finger DNA-binding domain (Yang et al., 2008a). ZKSCAN3 belongs to the ZKSCAN family, which is characterized by the Krüppel-associated box (KRAB) domain (Sobocinska et al., 2021). The ZKSCAN family can function as oncogenes or tumor suppressor genes (Sobocinska et al., 2021). A knockout study in mice shows that ZKSCAN3 is dispensable for embryonic development. ZKSCAN3-null mice can be born and grow normally (Pan et al., 2017). However, existing evidence suggests ZKSCAN3 may function in cancer. ZKSCAN3 is overexpressed in various cancer types (Cho et al., 2022; Kim et al., 2016; Yang et al., 2008a; Yang et al., 2008b). Potential involvement of ZKSCAN3 was reported in colorectal cancer (Cho et al., 2022; Kim et al., 2016; Yang et al., 2008a; Yang et al., 2008b), liver cancer (Li et al., 2020), multiple myeloma (Yang et al., 2011), prostate cancer (Zhang et al., 2012), bladder cancer (Kawahara et al., 2016), breast cancer (Chi et al., 2018), cervical cancer (Lee et al., 2018), and gastric cancer (Takano et al., 2020). However, the underlying mechanisms remain incompletely characterized and merit further investigation. ZKSCAN3 has both transcription activation and repression activities. On one hand, ZKSCAN3 binds and activates certain pro-tumor genes. In colorectal cancer and liver cancer, ZKSCAN3 promotes the expression of integrin-β4 (Li et al., 2020; Yang et al., 2008b). In multiple myeloma, ZKSCAN3 promotes expression of cyclin-D2 (Yang et al., 2011). On the other hand, ZKSCAN3 may repress gene expression. ZKSCAN3 was reported to inhibit the expression of autophagic genes (Chauhan et al., 2013). The autophagy suppressive role of ZKSCAN3 may be limited to tumor cells as no apparent abnormality in autophagy was seen in ZKSCAN3-null mice (Pan et al., 2017). In summary, past studies have revealed that ZKSCAN3 has a cancer-specific function and regulates the expression of cancer-related genes in a cancer type-specific manner. However, whether and how ZKSCAN3 has a role in ovarian cancer remains unknown.

In this study, we identify that ZKSCAN3 is overexpressed in ovarian cancer by analyzing a large-scale proteomic study of ovarian cancer. We show that knocking down ZKSCAN3 significantly retards ovarian cancer cell proliferation. To decipher the underlying mechanism, we characterize the effect of ZKSCAN3 on gene expression with RNA-Seq and chromatin immunoprecipitation (ChIP)-Seq. We identify HSPB1 as a direct target gene of ZKSCAN3, which encodes heat shock protein 27kD. We find that HSPB1 is required for the regulation of cell proliferation by ZKSCAN3 in ovarian cancer cells. Collectively, this study uncovers a functional ZKSCAN3-HSPB1 axis critical for ovarian cancer cell proliferation.

## 2 Results

### 2.1 ZKSCAN3 promotes ovarian cancer cell proliferation

**FIGURE 1:**
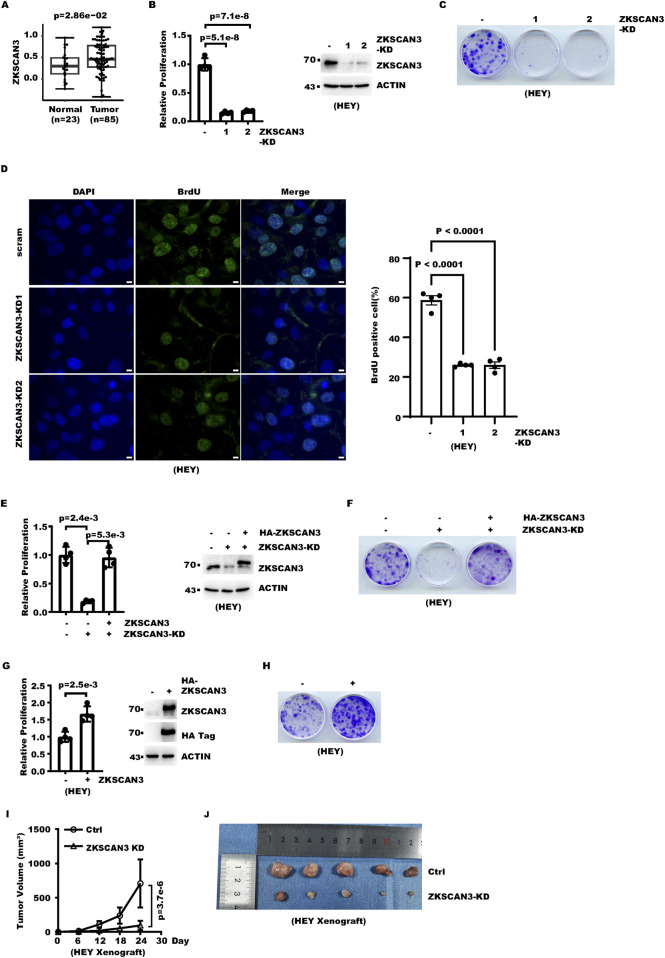
ZKSCAN3 promotes ovarian cancer cell proliferation. **(A)** The box plot shows the ZKSCAN3 protein levels in 23 normal ovarian samples *versus* 85 ovarian tumor samples. Data are from CPTAC (CPTAC PDC000110). **(B)** ZKSCAN3 was knocked down in ovarian cancer cells with lentivirus-expressed shRNA. Cell proliferation of control and ZKSCAN3-KD cells over 4 days was measured with cell counting. **(C)** ZKSCAN3 was knocked down in ovarian cancer cells with lentivirus-expressed shRNA. Each 3.5-cm dish was seeded with 1,000 cells. Cells were fixed with crystal violet 12 days later. **(D)** Control or ZKSCAN3-knockdown cells were labeled with BrdU and then immunostained with an anti-BrdU antibody. Scale bars denote 10 μm. **(E)** ZKSCAN3 was knocked down in ovarian cancer cells with lentivirus-expressed shRNA. An shRNA-resistant ZKSCAN3 cassette was rescue-expressed with lentivirus. Cell proliferation over 4 days was measured with cell counting. **(F)** ZKSCAN3 was knocked down in ovarian cancer cells with lentivirus-expressed shRNA. An shRNA-resistant ZKSCAN3 cassette was rescue-expressed with lentivirus. Each 1, 3.5-cm dish was seeded with 1,000 cells. Cells were fixed with crystal violet 12 days later. **(G)** ZKSCAN3 was stably overexpressed in ovarian cancer cells with lentivirus transduction. Cell proliferation over 5 days was measured with cell counting. **(H)** ZKSCAN3 was stably overexpressed in ovarian cancer cells with lentivirus transduction. Each 3.5-cm dish was seeded with 1,000 cells. Cells were fixed and stained with crystal violet 12 days later. **(I)** 5 × 10^6^ control or ZKSCAN3-KD cells were inoculated into immunodeficient mice. Shown are the growth curves of the xenograft. **(J)** Shown are photos of the xenograft collected from mice as described in **(H)** after mice were sacrificed.

We first asked whether ZKSCAN3 is differentially expressed between ovarian cancer samples and normal samples. We analyzed public proteomic data for ovarian cancer patients in the Clinical Proteomic Tumor Analysis Consortium (CPTAC) database. The analysis showed that ZKSCAN3 protein levels were higher in cancer samples at a statistically significant (p = 0.02) level (Figure 1A). Cell proliferation is a key indicator of tumor cell intrinsic effects. We examined whether ZKSCAN3 affected ovarian cancer cell proliferation. We knocked down ZKSCAN3 with lentivirus-expressed shRNA in different ovarian cancer cells, including HEY, A2780, and CaOV3. After selecting against non-transduced cells using antibiotics, we analyzed cell proliferation with two methods. First, we measured the change in the cell number with time. The result showed that ZKSCAN3 knockdown (KD) significantly reduced cell proliferation during the 4-day period (Figure 1B; Supplementary Figure S1A).

Second, we measured how ZKSCAN3 affected the ability of cells to form colonies. The result showed that ZKSCAN3 knockdown significantly inhibited ovarian cancer cell clonogenesis (Figure 1C; Supplementary Figure S1B). As a marker for active proliferation, 5-bromo-2'-deoxyuridine (BrdU) incorporation into cellular DNA was measured. The result showed ZKSCAN3 knockdown decreased BrdU incorporation (Figure 1D; Supplementary Figure S1C). Moreover, we found ZKSCAN3-KD inhibited cell proliferation synergistically with the chemotherapy drug oxaliplatin (Supplementary Figure S1D). In addition, we analyzed whether ZKSCAN3-KD affected cellular motility. Transwell and wound healing assays showed ZKSCAN3-KD reduced cell migration (Supplementary Figures S1E, F).

We next focused on the effect of ZKSCAN3 on cellular proliferation. To further confirm that the inhibitory effect on cell proliferation was specifically caused by ZKSCAN3-KD rather than an off-target effect, we rescue expressed an shRNA-resistant ZKSCAN3 expression cassette in ZKSCAN3-KD cells. Cell counting and colony formation assays showed that cell proliferation was indeed restored (Figures 1E,F; Supplementary Figures S1G, H), which demonstrated that the decreased cell proliferation in ZKSCAN3-KD cells was specifically due to reduced ZKSCAN3 level rather than an unanticipated off-target effect. We next examined whether ZKSCAN3 overexpression might promote cell proliferation. We stably expressed ZKSCAN3 in HEY and A2780 cells with lentivirus transduction followed by antibiotic selection. Cell counting showed that ZKSCAN3 overexpression increased cell proliferation (Figure 1G; Supplementary Figure S1I). Colony formation assays also confirmed ZKSCAN3 overexpression increased cell clonogenesis (Figure 1H; Supplementary Figure S1J).

We further examined whether ZKSCAN3 promoted ovarian cancer growth with a mouse xenograft model. Both control and ZKSCAN3-KD cells were inoculated into immunodeficient mice. The result showed ZKSCAN3-KD significantly decreased tumor growth (Figure 1I). At the end point, tumors from ZKSCAN3-KD cells were significantly smaller than the control (Figure 1J). Collectively, these data show ZKSCAN3 promotes ovarian cancer cell proliferation.

### 2.2 ZKSCAN3 regulates gene expression in ovarian cancer

**FIGURE 2:**
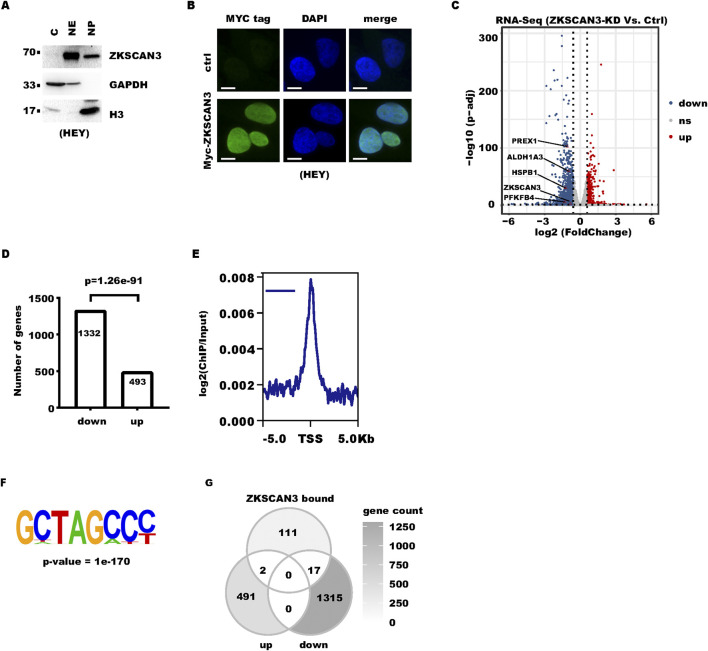
ZKSCAN3-regulated cancer-associated pathways: **(A)** Cells were fractionated into cytoplasm (c), nuclear extract (NE), and nuclear pellet (NP) fractions. Equal amounts of these fractions were analyzed by Western blot (WB). GAPDH and Histone H3 were included as markers for the cytoplasm and nuclei, respectively. **(B)** Myc-tag ZKSCAN3 was stably expressed in HEY cells with lentivirus transduction followed by antibiotic selection. Cells were subject to immunofluorescence analysis with a Myc-tag antibody. Nuclei were counterstained with DAPI. Scale bars denote 10 μm. **(C)** The volcano plot summarizes the gene differential expression between control and ZKSCAN3-KD HEY cells. “Up” denotes genes upregulated in “ZKSCAN3-KD” cells, “Down” denotes genes upregulated in “ZKSCAN3-KD” cells, and “ns” denotes genes without significant changes. Genes with fold of change larger than 1.5 and p-adjusted less than 0.05 were considered as “significant.” **(D)** The bar plot shows the number of downregulated (down) and upregulated (up) genes after ZKSCAN3-KD. **(E)** Profile plot shows the distribution of the ZKSCAN3 ChIP-seq signal around TSS. **(F)** The image shows the top enriched motif in ZKSCAN3-bound loci. **(G)** The Venn diagram summarizes the number of genes significantly changed by ZKSCAN3-KD, genes with ZKSCAN3 enrichment, and genes in both categories.

We next asked how ZKSCAN3 promoted ovarian cancer cell proliferation. It was reported that ZKSCAN3 subcellular localization is subject to regulation by nutrient status in certain cell types (Chauhan et al., 2013; Li et al., 2016). Therefore, ZKSCAN3 can mainly localize to the cytoplasm under starvation (Chauhan et al., 2013). We characterized ZKSCAN3 localization in HEY ovarian cancer cells. Fractionation showed ZKSCAN3 mainly localized to the cell nucleus (Figure 2A). To make sure of the signal specificity, the Myc-tag antibody was also used for immunofluorescence for stably expressed Myc-tagged ZKSCAN3. The result supported ZKSCAN3 being mainly localized to the cell nucleus under normal culture conditions (Figure 2B).

Nuclear localization indicated ZKSCAN3 might function through transcription regulation. To decipher the transcriptional network targeted by ZKSCAN3, we performed RNA-Seq to compare the transcriptomes of ZKSCAN3-KD cells and control cells. At approximately 50% ZKSCAN3 knockdown efficiency, 1,825 genes were significantly regulated (change fold ≥ 1.5 and p-adjust <0.05) by ZKSCAN3-KD, among which 493 were upregulated and 1,332 were downregulated (Figure 2C). The fact that ZKSCAN3-KD caused a significant change in approximately 10% of all protein-coding genes indicated that ZKSCAN3 might have a significant role in ovarian cancer biology. Statistically, significantly more genes are downregulated than upregulated (Figure 2D). To validate the reliability of the RNA-Seq result, we picked three genes, PFKFB4, ALDH1A3, and PREX1, for validation with real-time RT-PCR. The result of RT-qPCR well recapitulated that of RNA sequencing (Supplementary Figure S2).

To identify genes that were directly targeted by ZKSCAN3, we stably expressed HA-tagged ZKSCAN3 in HEY cells and performed ChIP-sequencing with an HA-tag antibody. Consistent with its role as a transcription factor, ZKSCAN3 binds preferentially to the gene transcription initiation region (Figure 2E). Among the ChIP-seq peaks, a GCTAGCCC motif was enriched (Figure 2F). Among genes with ZKSCAN3 enrichment signals, 19 overlapped with those differentially expressed upon ZKSCAN3-KD (Figure 2G). Seventeen of these genes were downregulated in ZKSCAN3-KD cells, and two were upregulated, which indicated ZKSCAN3 played a role in activating gene expression in ovarian cancer cells. In addition, most genes whose expression is regulated by ZKSCAN3 do not seem to be targeted by ZKSCAN3, which indicates these genes are regulated by ZKSCAN3 indirectly. Collectively, these data suggested ZKSCAN3 regulated the expression of cancer-related genes in ovarian cancer cells.

### 2.3 ZKSCAN3 promoted HSPB1 expression

**FIGURE 3:**
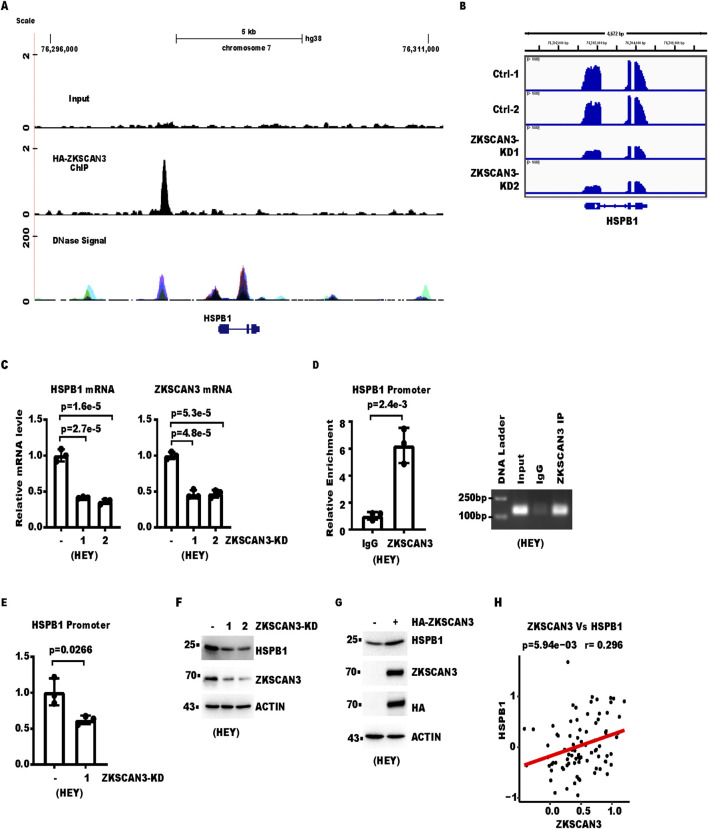
HSPB1 expression is regulated by ZKSCAN3. **(A)** Shown is a snapshot of the UCSC genome browser that illustrates the ChIP-seq signals from Input and ZKSCAN3-ChIP at the HSPB1 locus. DNase signal was from ENCODE. **(B)** Shown is a snapshot of Integrative Genomics Viewer, which illustrates the RNA-Seq signals at the HSPB1 locus. **(C)** The HSPB1 mRNA levels in the control and ZKSCAN3-KD cells were analyzed with real-time RT-PCR. ZKSCAN3 mRNA was included to show knockdown efficiency. mRNA levels presented were normalized to ACTB (β-actin). **(D)** Cells were analyzed with ChIP. Immunoprecipitation was done with the ZKSCAN3 antibody with IgG as the control. The left panel shows the result of real-time PCR, and the right panel shows the agarose gel image for conventional PCR products. **(E)** Control or ZKSCAN3-KD cells were analyzed with ChIP followed by real-time PCR. Immunoprecipitation was done with the ZKSCAN3 antibody. IgG was included as a control. **(F)** ZKSCAN3 was knocked down with lentivirus-expressed shRNA. The whole-cell lysate (WCL) was then analyzed by Western blot (WB). **(G)** ZKSCAN3 was stably expressed in HEY ovarian cancer cells with lentivirus transduction followed by antibiotic selection. The whole-cell lysate (WCL) was then analyzed by Western blot (WB). **(H)** Shown is linear regression for protein levels of ZKSCAN3 and HSPB1 in 85 ovarian cancer patients from the CPTAC (PDC000110).

Among genes that had ZKSCAN3 enrichment and were significantly regulated by ZKSCAN3, HSPB1 was of particular interest. HSPB1 encodes heat shock protein 27kD (HSP27), which was reported to affect various aspects of cancer progression (Arrigo, 2017). In the ChIP-seq experiment, the HSPB1 genomic locus contains significant enrichment of ZKSCAN3 (Figure 3A). Cross-referencing with the ENCODE result shows that the ZKSCAN3 peak overlaps with a DNase I hypersensitive region, a marker for active regulatory DNA elements (Figure 3A). RNA-seq revealed a significant decrease in HSPB1 mRNA level after ZKSCAN3 knockdown of approximately 50% (Figure 3B), which was confirmed by RT-qPCR after ZKSCAN3 knockdown with two independent shRNAs (Figure 3C). Furthermore, we confirmed ZKSCAN3 enrichment at HSPB1 with ChIP followed by qPCR in both HEY and A2780 ovarian cancer cell lines (Figure 3D; Supplementary Figure S3A). The signal specificity was validated by its being significantly higher than that in immunoglobulin G (IgG) immunoprecipitate (Figure 3D; Supplementary Figure S3A). To further confirm the signal specificity, we compared the ChIP signals in ZKSCAN3-KD *versus* control cells. The result showed that the ZKSCAN3 signal at HSPB1 was decreased in ZKSCAN3-KD cells (Figure 3E).

We next examined whether the HSPB1 protein level was also regulated by ZKSCAN3. Western blot showed ZKSCAN3-KD indeed decreased the HSPB1 protein level in three different ovarian cancer cell lines (Figure 3F; Supplementary Figure S3B), which was consistent with the change in mRNA level. Then, we examined the HSPB1 level in ZKSCAN3-overexpression cells. The result showed ZKSCAN3 overexpression increased HSPB1 protein level (Figure 3G; Supplementary Figure S3C). To examine whether the relationship between ZKSCAN3 and HSPB1 was also present in ovarian cancer patient samples, we analyzed the CPTAC ovarian cancer cohort, which revealed a statistically significant positive correlation between HSPB1 protein level and ZKSCAN3 protein level (p < 0.01) (Figure 3H). Collectively, these data show HSBP1 is a target gene of ZKSCAN3 and is upregulated by ZKSCAN3.

### 2.4 HSPB1 contributes to ZKSCAN3-mediated promotion of cell proliferation

**FIGURE 4:**
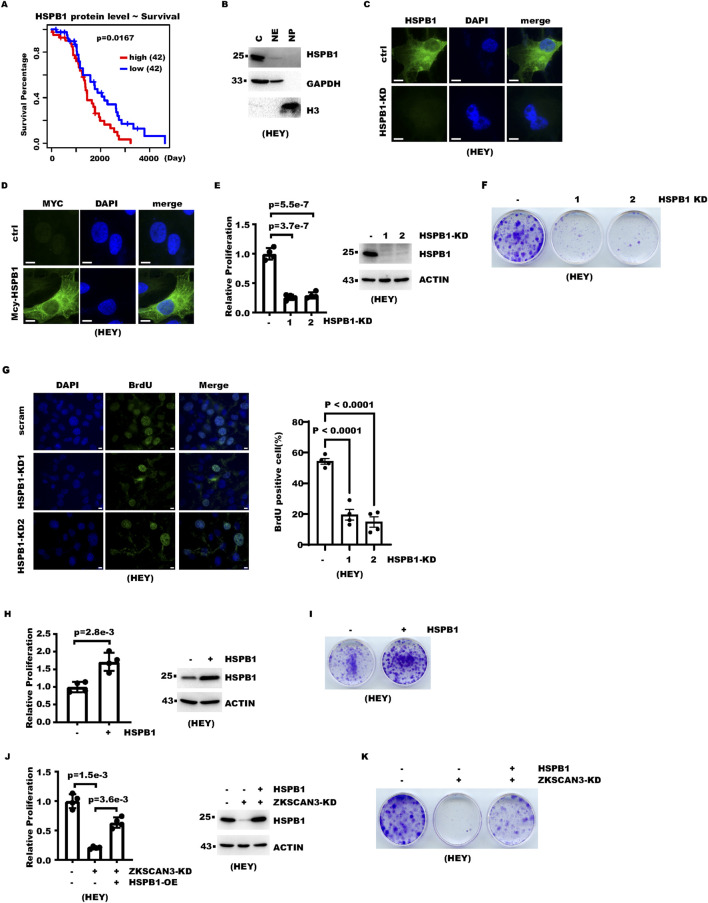
HSPB1 contributes to ZKSCAN3-mediated promotion of cell proliferation. **(A)** Survival analysis for 84 patients in the CPTAC ovarian cancer cohort. Patients were divided into “HSPB1 high” and “HSPB1 low” groups based on HSPB1 protein levels as determined by mass spectrometry. Median HSPB1 level was used as a threshold. **(B)** Cells were fractionated into cytoplasm (c), nuclear extract (NE), and nuclear pellet (NP) fractions. Equal amounts of these fractions were analyzed with WB. GAPDH and histone H3 were included as markers for cytoplasm and nuclei, respectively. **(C)** Control and HSPB1-KD cells were subject to immunofluorescence analysis with the HSPB1 antibody. Nuclei were counterstained with DAPI. Scale bars denote 10 μm. **(D)** Myc-tag HSPB1 was stably expressed in HEY cells with lentivirus transduction followed by antibiotic selection. Cells were then subject to immunofluorescence analysis with Myc-tag antibody. Nuclei were counterstained with DAPI. Scale bars denote 10 μm. **(E)** HSPB1 was knocked down in ovarian cancer cells with lentivirus-expressed shRNA. Cell proliferation over 4 days was measured with cell counting. **(F)** HSPB1 was knocked down in ovarian cancer cells with lentivirus-expressed shRNA. Each 3.5-cm dish was seeded with 1,000 cells. Cells were fixed with crystal violet 12 days later. **(G)** Control or HSPB1-knockdown cells were labeled with BrdU and then subject to immunostaining with anti-BrdU antibody. The left panel shows representative images, while the right panel shows the statistical results for four independent images. Scale bars denote 10 μm. **(H)** HSPB1 was overexpressed in ovarian cancer cells. Cell proliferation over 5 days was measured with cell counting. **(I)** HSPB1 was overexpressed in ovarian cancer cells. Each 3.5-cm dish was seeded with 1,000 cells. Cells were fixed with crystal violet 12 days later. **(J)** HSPB1 was expressed with lentivirus transduction in ZKSCAN3-KD cells. Cell proliferation over 4 days was then measured with cell counting. **(K)** HSPB1 was rescue expressed with lentivirus in ZKSCAN3-KD cells. Each 3.5-cm dish was seeded with 1,000 cells. Cells were fixed with crystal violet 12 days later.

We then analyzed the potential contribution to ovarian cancer cell proliferation. Survival analysis showed that a higher HSPB1 protein level correlated with poorer survival (Figure 4A). Subcellular fractionation and immunostaining showed that both endogenous and exogenous HSPB1 are mainly localized to the cell cytoplasm (Figures 4B–D). Both cell counting and colony formation assays showed that HSPB1 knockdown significantly inhibited proliferation in ovarian cancer cells (Figures 4E,F; Supplementary Figures S1A, B). A BrdU incorporation assay also showed that HSPB1 knockdown decreased cell proliferation (Figure 4G; Supplementary Figure S4C). Consistently, proliferation assays showed HSPB1 overexpression increased cell proliferation (Figures 4H,I; Supplementary Figures S1D, E).

After showing that HSPB1 promoted cell proliferation, we next asked whether the regulation of HSPB1 contributed to proliferation promotion by ZKSCAN3. In ZKSCAN3-KD cells, we expressed exogenous HSPB1 to bring the HSPB1 level comparable to that of the control cells. The result showed that proliferation of ZKSCAN3-KD cells was indeed partially restored (Figures 4J,K; Supplementary Figures S1F, G). In summary, these data establish HSPB1 as an effector of ZKSCAN3 that promotes ovarian cancer cell proliferation.

## 3 Discussion

### 3.1 ZKSCAN3 promotes ovarian cancer proliferation

It was unclear whether ZKSCAN3 plays a role in ovarian cancer. Analysis of patient sample proteomic quantitation data indicates that the ZKSCAN3 protein is overexpressed in ovarian cancer. Following functional studies in cell lines and mouse models, ZKSCAN3 was identified as having a significant impact on cell proliferation. Yet, ZKSCAN3 knockout does not change the overall survival in mice (Pan et al., 2017). Therefore, it is reasonable to postulate that ZKSCAN3 and its downstream pathway could be potential targets for an ovarian cancer therapeutic target.

### 3.2 ZKSCAN3 increases HSPB1 expression

In this study, we identify HSPB1 as a target gene of ZKSCAN3 with an unbiased functional genomics study. ZKSCAN3 not only upregulates HSPB1 expression but also binds close to its genomic locus. Our RNA-Seq and ChIP-seq experiments show, among those genes regulated by ZKSCAN3-KD and meanwhile bound by ZKSCAN3, most genes are downregulated, which supports a gene activation role of ZKSCAN3. Previous studies show ZKSCAN3 has both transcription activation and repression activities. However, the underlying mechanism for activation or repression remains enigmatic. Past studies on other bipartite proteins indicated that their output on transcription may be dependent on the epigenetic machinery they actually interact with (Sobocinska et al., 2021). Whether and how epigenetic machinery contributes to ZKSCAN3-mediated transcription regulation merits future investigation.

### 3.3 HSPB1 is a mediator of ZKSCAN3 function

HSPB1 is a low-molecular-weight heat shock response protein. Its expression can be induced by heat shock through the activity of master heat shock responsive transcription factor HSF1 (Carra et al., 2017). Consistently, it contributes to proper protein folding under heat stress. Importantly, it carries out multiple other functions related to cancer pathophysiology. Inhibiting HSPB1 leads to decreased cell proliferation and/or cell death (Park et al., 2018; Peng et al., 2023; Sun et al., 2015; Wang et al., 2016). HSPB1 also affects ovarian cancer response to therapy and serves as an independent prognostic factor (Geisler et al., 2004). Despite its pleiotropic functions in cancer biology, little was known about its expression regulation except that it is regulated by heat shock (Carra et al., 2017). In this study, we identify that HSPB1 transcription is regulated by another transcription factor, ZKSCAN3. This mechanism does not require heat shock and contributes to the basal level of HSPB1 expression. In addition, we find that the regulation of HSPB1 contributes to ZKSCAN3 function in ovarian cancer. Restoring HSPB1 expression significantly restores the cell proliferation defect in ZKSCAN3-KD cells. Findings in this study collectively suggest a functional ZKSCAN3-HSPB1 axis in ovarian cancer.

## 4 Methods

### 4.1 Western blot

Whole cell lysates were generated using 1× SDS lysis buffer (62.5 mM Tris-HCl, pH 6.8, 2% SDS, 10% glycerol, 5% β-mercaptoethanol) unless specified otherwise. Lysates underwent sonication followed by heat denaturation (99 °C, 5 min) and clarification via centrifugation (12,000 × g, 10 min). Protein quantification was performed using the BCA assay (Beyotime, P0012), with 20–50 μg protein loaded per SDS-PAGE well. Sample volumes were normalized by adding 1× SDS loading buffer (base lysis buffer +0.002% bromophenol blue). Immunoprecipitated proteins were resuspended in 1× SDS loading buffer, while *in vitro* reaction products were mixed with 5× SDS loading buffer (312.5 mM Tris-HCl, pH 6.8, 10% SDS, 30% glycerol, 25% β-mercaptoethanol, 0.01% bromophenol blue) to achieve a 2% final SDS concentration. Electrophoresis was conducted using a Bio-Rad Mini-PROTEAN Tetra system, followed by PVDF membrane transfer (Millipore IPVH00010) with a Bio-Rad Trans-Blot module. Membranes were blocked with 5% non-fat milk in TBST (10 mM Tris, pH 7.4, 200 mM NaCl, 0.05% Tween-20) prior to overnight incubation with primary antibodies at 4 °C. After thorough washing, membranes were probed with HRP-conjugated secondary antibodies (3 h, RT) and visualized through enhanced chemiluminescence using a Bio-Rad ChemiDoc system. Antibodies used are as follows: ZKSCAN3 (Santa Cruz #sc-515285); HSPB1 (Proteintech #PTG_18284-1-AP); GAPDH (ABclonal #AC033); β-ACTIN (ABclonal #AC026); HA-tag (Cell Signaling Technology #3724); Histone H3 (Abcam #ab1791); secondaries (Jackson ImmunoResearch 111-035-003 and 115-035-003).

### 4.2 Cell culture

Caov3 (CL-0055) cells were from Wuhan Pricella Biotechnology Co., Ltd. (Wuhan, China). HEK-293T cells (human embryonic kidney cells) were sourced from Dr. Shuguo Sun’s laboratory at Huazhong University of Science and Technology. A2780, HEY, and Caov3 cells were cultured in DMEM (Gibco #12800082) containing 10% FBS (PAN #ST30-3302). HEK-293T cells were maintained in DMEM (Gibco #12800082) containing 10% FBS (PAN #ST30-3302). All cultures were incubated at 37 °C under 5% CO_2_.

### 4.3 Cell proliferation

HEY and A2780 cells were plated at 5 × 10^5^ cells per 35-mm culture dish and maintained under experimental conditions with bi-daily subculturing. Cellular proliferation was quantified using an automated cell counting system. Where indicated, cells were treated with 0.2 μM oxaliplatin (MCE HY-17371) for 3 days.

### 4.4 Cellular fractionation

Nuclear-cytoplasmic separation was based on established protocols (Song et al., 2021). PBS-washed cells were harvested and resuspended in hypotonic buffer (5× pellet volume: 10 mM HEPES, pH 7.9, 10 mM KCl, 0.5 mM DTT, 0.5 mM PMSF, 1.5 mM MgCl_2_), then incubated on ice for 15 min. NP-40 was added to a 0.2% final concentration, followed by 10 s vortex mixing. Cytoplasmic–nuclear separation was achieved by centrifugation (5,000 × g, 30 sec), with the supernatant retained as the cytoplasmic fraction. For nuclear extraction, pellets were resuspended in 1.5× volume of low-salt buffer (20 mM HEPES, pH 7.9, 150 mM NaCl, 0.5 mM DTT/PMSF, 1.5 mM MgCl_2_) and combined with 1.5× volume high-salt buffer (20 mM HEPES, pH 7.9, 650 mM NaCl, 0.5 mM DTT/PMSF, 1.5 mM MgCl_2_). After a 30-min rotation (4 °C) and centrifugation (15,000 × g, 15 min, 4 °C), the supernatant constituted the nuclear extract. Residual nuclear debris was sonicated in SDS lysis buffer and constituted the nuclear pellet.

### 4.5 Colony formation

Cells were harvested via trypsinization and resuspended in fresh medium. A standardized inoculum of 1 × 10^3^ cells per 35-mm dish was plated and cultured for 12 days with medium replacement every 3 days. Post-incubation, monolayers were rinsed twice with PBS and fixed in methanol (5 min). Colonies were stained with 0.5% methanol-based crystal violet (10 min), followed by five distilled water washes to remove unbound dye. Air-dried dishes were digitally archived using an Epson Perfection V550 scanner.

### 4.6 Nude mice experiment

All procedures complied with institutional animal care guidelines approved by the Ethics Committee of Tongji Medical College, Huazhong University of Science and Technology. Mice were acclimatized for ≥7 days in ventilated cages under controlled conditions (21 °C ± 1 °C, 12-h light/dark cycle) with *ad libitum* access to food/water. For xenograft studies, 6–7-week-old female NU/NU mice (Charles River) received subcutaneous flank injections of 5 × 10^6^ cells suspended in 100 μL PBS-Matrigel (1:1; BD Biosciences #354248). Tumor dimensions were monitored biweekly/triweekly using calipers, with volume calculated as 0.5 × length × width^2^.

### 4.7 Immunofluorescence

Cells were fixed with 4% paraformaldehyde (15 min, RT) and permeabilized using 0.5% Triton X-100 in PBS (4 °C). After blocking with 1% BSA, samples were incubated with primary antibodies against Myc tag (Proteintech 16286-1-AP) or HSPB1 (Proteintech #18284-1-AP). Following three washes with PBST (0.2% Tween-20), Alexa fluor-conjugated secondary antibodies (Jackson ImmunoResearch #711-545-152) were applied for 1 h. Nuclei were counterstained with DAPI (5 min, RT), and coverslips were mounted using antifade medium (Abcam AB104135). Imaging was performed on a Zeiss Axiovert A1 microscope (63× oil immersion lens) equipped with a Retiga R6 camera. Image analysis utilized Fiji/ImageJ (NIH).

### 4.8 BrdU staining

Cells were first treated with 10 μM BrdU (Targetmol #T6794) for 2 h and then fixed with 4% paraformaldehyde. After fixation, cells were permeabilized with 0.5% Triton-X100. Subsequently, cells were first incubated with 1 M HCl for 30 min to denature the DNA, followed by incubation with 0.1 M sodium borate buffer and then blocking with 1% BSA. Cells were then stained using a procedure similar to that described in the “Immunofluorescence” section. The BrdU antibody used for BrdU detection was from Proteintech (#66241-1-Ig).

### 4.9 Transwell assay

A total of 1 × 10^5^ cells were seeded into the top chamber of a Transwell insert with 8.0-μm pores (Corning #3422) filled with serum-free medium, while the bottom chamber was filled with normal medium. Cells were allowed to migrate through the membrane for 24 h. Subsequently, non-migrated cells in the top chamber were removed with a cotton swab, and the cells on the bottom of the membrane were fixed and stained with crystal violet. Images were taken under an OLYMPUS IX73 fluorescent microscope.

### 4.10 Wound healing assay

Cells were seeded into 3.5-cm dishes. Scratch wounds were created using an end-cut 200-μL pipette tip. Subsequently, images were taken at different time points under an OLYMPUS IX73 fluorescent microscope.

### 4.11 Chromatin immunoprecipitation

Chromatin immunoprecipitation assays were conducted as described previously (Song et al., 2021). Cells were crosslinked with 2.5 mM disuccinimidyl glutarate (Aladdin #D304655) for 45 min and 1% formaldehyde (Sigma #F8775) under gentle agitation for another 10 min, followed by quenching with 0.125 M glycine. After ice-cold PBS washes, cells were harvested by mechanical scraping and resuspended in hypotonic buffer A (10 mM HEPES, pH 7.9, 1.5 mM MgCl_2_, 10 mM KCl, 0.5% NP-40, 0.5 mM DTT) for 10 min on ice. Chromatin was fragmented using a BioRuptor sonicator (Diagenode) in lysis buffer (50 mM Tris-Cl, pH 8.0, 5 mM EDTA, 0.2% SDS). Clarified lysates (15,000 × g, 10 min, 4 °C) were combined 1:1 with dilution buffer (300 mM NaCl, 2% Triton X-100) and incubated overnight with antibodies against HA (Santa Cruz #sc-7392) at 1 μg per 200-μL lysate. Immunocomplexes were captured using Protein G magnetic beads (CST 9006). After stringent washing, bound complexes were eluted in buffer (0.1 M NaHCO_3_, 1% SDS) at 65 °C with agitation (15 min). Reverse crosslinking occurred overnight at 65 °C, followed by DNA purification with a Quick PCR purification kit (Invitrogen, K310001). Quantitative PCR analysis was performed using primers for HSPB1, as detailed in Supplementary Table S1.

### 4.12 Total RNA extraction and reverse transcription

Cellular RNA was isolated using TRIzol reagent (Invitrogen #15596018) following standard protocols. RNA concentration was measured using a NanoDrop spectrophotometer (Thermo Scientific). For DNA removal, 1 μg RNA samples were treated with DNase I (Thermo Scientific #EN0521) according to manufacturer specifications. First-strand cDNA synthesis was performed using the Improm-II Reverse Transcription System (Toyobo #FSQ-101). Briefly, 1 μg DNase-treated RNA was combined with 0.5-μg random primer mix, heat-denatured at 65 °C (5 min), and then immediately cooled on ice. The reaction mixture was prepared by adding dNTPs, reverse transcriptase, and RNase inhibitor, followed by incubation at 37 °C for 15 min. Reactions were terminated through heat inactivation at 98 °C for 5 min.

### 4.13 Real-time quantitative PCR

qPCR amplification was conducted using commercially available SYBR Green master mixes (Toyobo ThunderBird QPK-201) in Bio-Rad-compatible 96-well plates (HSP9655/MSB1001) on a CFX Connect thermocycler. Primer sequences for quantitative PCR are detailed in Supplementary Table S1.

### 4.14 Lentivirus-mediated knockdown

shRNA systems were generated using pLKO vectors (Moffat et al., 2006). Oligonucleotides were hybridized and cloned into AgeI/EcoRI-digested pLKO-puro (constitutive) backbones. Sequence accuracy was confirmed via Sanger sequencing. Lentiviral particles were produced by co-delivering shRNA constructs with psPAX2 and pMD2. G packaging plasmids into HEK293T cells. Viral supernatants were harvested at 48 h, filtered (0.45 μm), and applied to target cells. For adherent cultures, viral suspensions were supplemented with 8 μg/mL polybrene (Sigma H9268) and directly added to the media. Transduced cells were expanded 24 h post-infection, followed by puromycin selection (1 μg/mL, InvivoGen ANT-PR-1) initiated 48 h after transduction.

### 4.15 Lentivirus-mediated gene overexpression or rescue-expression

The cDNA insert was ligated into the blasticidin-resistant pLenti-EF1a backbone using conventional restriction-based cloning (NEB restriction enzymes; Promega UC6711 T4 DNA ligase), with sequence fidelity confirmed by Sanger sequencing. Lentiviral particles were generated through co-transfection of HEK-293T cells with the transfer vector, psPAX2, and pMD2. G packaging plasmids. Viral supernatants were harvested 48 h post-transfection, filtered (0.45 μm, Millipore SLHP033RB), and applied to target cells. Resistance to shRNA was achieved by the synonymous mutation in the coding region. For adherent cell lines, viral suspensions were directly introduced to culture media containing 8 μg/mL polybrene (Sigma H9268). Transduced populations were selected using 10 μg/mL blasticidin (InvivoGen ANT-BL-1) for 3–7 days prior to functional assays.

### 4.16 ChIP-seq and analysis

Chromatin immunoprecipitation was conducted as outlined in the “Chromatin immunoprecipitation” section. HA-ZKSCAN3-expressing HEY cells were subjected to ChIP using anti-HA antibody (Santa Cruz #sc-7392). Library preparation and sequencing were outsourced to BGI (Shenzhen, China), employing the MGIEasy DNA Library Prep Kit (MGI, Shenzhen, China), following dA-tailing, adapter ligation, and size selection. PCR-amplified libraries underwent quality control prior to 150-bp paired-end sequencing on a BGI G400 system. Raw reads were first filtered with Trim-Galore and then aligned with bowtie2 (53) to the human hg38 genome assembly. Alignments with MAPQ >2 were kept, and genomic coverage in bigwig format (bin = 25 bp) was generated with deepTools BamCoverage (54). Reads coverage at HSPB1 was viewed with the UCSC genome browser or Integrative Genomics Viewer. Read coverage around a transcription start site (TSS) was calculated by deepTools ComputeMatrix (bin = 25 bp) and plotted with deepTools plotHeatmap.

### 4.17 RNA-Sseq and analysis

HEY cells were infected with pLKO-based lentiviruses encoding ZKSCAN3-targeting or non-targeting shRNAs, followed by 3-day puromycin selection. Total RNA was isolated using TRIzol (Invitrogen #15596018) per protocol guidelines. RNA sequencing was conducted by BGI Genomics (Shenzhen, China), involving mRNA enrichment via Oligo (dT) beads, fragmentation, and double-stranded cDNA synthesis with random priming. Libraries were prepared through dA-tailing, adapter ligation, and PCR amplification, followed by quality assessment on an Agilent 2100 Bioanalyzer. DNA nanoballs generated via Phi29 polymerase amplification underwent 150-bp paired-end sequencing on the MGISEQ-2000RS platform. Raw reads were quality-trimmed (Trim-Galore) and aligned to the GENCODE v39-annotated hg38 genome using STAR. Low-quality alignments (MAPQ <10) and PCR duplicates (RmDup-filtered) were excluded. Coverage tracks were generated via deepTools bamCoverage, while featureCounts quantified gene-level reads. Differential expression analysis utilized DESeq2 with default parameters.

### 4.18 Statistical analysis

For cell proliferation, the number of cells was measured with four biological replicates, and p-values were calculated with one-way ANOVA with correction for multiple comparisons. In xenograft studies, tumor growth curves displayed group-wise SD (n = 6 mice/group). Statistical comparisons employed two-way ANOVA, with GraphPad Prism v8.0 calculating significance thresholds (p < 0.05 considered statistically significant). Fisher’s exact test was performed with the “fisher.test” in R 4.3.3. Quantitative PCR data were analyzed and graphically represented using Excel (Microsoft) and GraphPad Prism v8.0, with error bars indicating standard deviation (SD) of triplicates. To analyze the linear correlation between HSPB1 and ZKSCAN3, protein expression data for ovarian cancer samples were downloaded from CPTAC. The correlation coefficients and p-values were calculated with R 4.3.3. For survival analysis, patients in the CPTAC PDC000114 cohort were divided into two groups based on their HSPB1 protein level in the ovarian cancer samples, with the median level as the threshold. Then, survival analysis was done with the “Surv” package in R 4.3.3.

## Data Availability

The datasets presented in this study are deposited in the GEO repository, accession number GSE248519.

## References

[B1] Arrigo A. P. (2017). Mammalian HspB1 (Hsp27) is a molecular sensor linked to the physiology and environment of the cell. Cell Stress Chaperones 22, 517–529. 10.1007/s12192-017-0765-1 28144778 PMC5465029

[B2] Carra S. Alberti S. Arrigo P. A. Benesch J. L. Benjamin I. J. Boelens W. (2017). The growing world of small heat shock proteins: from structure to functions. Cell Stress Chaperones 22, 601–611. 10.1007/s12192-017-0787-8 28364346 PMC5465036

[B3] Chauhan S. Goodwin J. G. Chauhan S. Manyam G. Wang J. Kamat A. M. (2013). ZKSCAN3 is a master transcriptional repressor of autophagy. Mol. Cell 50, 16–28. 10.1016/j.molcel.2013.01.024 23434374 PMC3628091

[B4] Chen W. Zheng R. Baade P. D. Zhang S. Zeng H. Bray F. (2016). Cancer statistics in China, 2015. CA a Cancer J. Clin. 66, 115–132. 10.3322/caac.21338 26808342

[B5] Chi Y. Xu H. Wang F. Chen X. Shan Z. Sun Y. (2018). ZKSCAN3 promotes breast cancer cell proliferation, migration and invasion. Biochem. Biophys. Res. Commun. 503, 2583–2589. 10.1016/j.bbrc.2018.07.019 30049438

[B6] Cho Y. E. Kim J. H. Che Y. H. Kim Y. J. Sung J. Y. Kim Y. W. (2022). Role of the WNT/β-catenin/ZKSCAN3 pathway in regulating chromosomal instability in Colon cancer cell lines and tissues. Int. J. Mol. Sci. 23, 9302. 10.3390/ijms23169302 36012568 PMC9409321

[B7] Geisler J. P. Tammela J. E. Manahan K. J. Geisler H. E. Miller G. A. Zhou Z. (2004). HSP27 in patients with ovarian carcinoma: still an independent prognostic indicator at 60 months follow-up. Eur. J. Gynaecol. Oncol. 25, 165–168. 10.12892/ejgo200402165 15032273

[B8] George A. Kaye S. Banerjee S. (2017). Delivering widespread BRCA testing and PARP inhibition to patients with ovarian cancer. Nat. Rev. Clin. Oncol. 14, 284–296. 10.1038/nrclinonc.2016.191 27958297

[B9] Gourley C. Bookman M. A. (2019). Evolving concepts in the management of newly diagnosed epithelial ovarian cancer. J. Clin. Oncol. 37, 2386–2397. 10.1200/JCO.19.00337 31403859

[B10] Kawahara T. Inoue S. Ide H. Kashiwagi E. Ohtake S. Mizushima T. (2016). ZKSCAN3 promotes bladder cancer cell proliferation, migration, and invasion. Oncotarget 7, 53599–53610. 10.18632/oncotarget.10679 27447553 PMC5288208

[B11] Kim C. W. Roh S. A. Tak K. H. Koh B. M. Ha Y. J. Cho D. H. (2016). ZKSCAN3 facilitates liver metastasis of colorectal cancer associated with CEA-Expressing tumor. Anticancer Res. 36, 2397–2406. 27127149

[B12] Lee S. Cho Y. E. Kim J. Y. Park J. H. (2018). ZKSCAN3 upregulation and its poor clinical outcome in uterine cervical cancer. Int. J. Mol. Sci. 19, 2859. 10.3390/ijms19102859 30241382 PMC6213532

[B13] Li Y. Xu M. Ding X. Yan C. Song Z. Chen L. (2016). Protein kinase C controls lysosome biogenesis independently of mTORC1. Nat. Cell Biol. 18, 1065–1077. 10.1038/ncb3407 27617930

[B14] Li J. Hao N. Han J. Zhang M. Li X. Yang N. (2020). ZKSCAN3 drives tumor metastasis *via* integrin β4/FAK/AKT mediated epithelial-mesenchymal transition in hepatocellular carcinoma. Cancer Cell Int. 20, 216. 10.1186/s12935-020-01307-7 32518525 PMC7275473

[B15] Moffat J. Grueneberg D. A. Yang X. Kim S. Y. Kloepfer A. M. Hinkle G. (2006). A lentiviral RNAi library for human and mouse genes applied to an arrayed viral high-content screen. Cell 124, 1283–1298. 10.1016/j.cell.2006.01.040 16564017

[B16] Nameki R. Chang H. Reddy J. Corona R. I. Lawrenson K. (2021). Transcription factors in epithelial ovarian cancer: histotype-specific drivers and novel therapeutic targets. Pharmacol. Ther. 220, 107722. 10.1016/j.pharmthera.2020.107722 33137377

[B17] Pan H. Yan Y. Liu C. Finkel T. (2017). The role of ZKSCAN3 in the transcriptional regulation of autophagy. Autophagy 13, 1235–1238. 10.1080/15548627.2017.1320635 28581889 PMC5529079

[B18] Park A. M. Tsunoda I. Yoshie O. (2018). Heat shock protein 27 promotes cell cycle progression by down-regulating E2F transcription factor 4 and retinoblastoma family protein p130. J. Biol. Chem. 293, 15815–15826. 10.1074/jbc.RA118.003310 30166342 PMC6187643

[B19] Peng S. Yin Y. Zhang Y. Zhu F. Yang G. Fu Y. (2023). FYN/TOPK/HSPB1 axis facilitates the proliferation and metastasis of gastric cancer. J. Exp. Clin. Cancer Res. 42, 80. 10.1186/s13046-023-02652-x 37016377 PMC10071617

[B20] Sobocinska J. Molenda S. Machnik M. Oleksiewicz U. (2021). KRAB-ZFP transcriptional regulators acting as oncogenes and tumor suppressors: an overview. Int. J. Mol. Sci. 22, 2212. 10.3390/ijms22042212 33672287 PMC7926519

[B21] Song T. Zou Q. Yan Y. Lv S. Li N. Zhao X. (2021). DOT1L O-GlcNAcylation promotes its protein stability and MLL-Fusion leukemia cell proliferation. Cell Rep. 36, 109739. 10.1016/j.celrep.2021.109739 34551297

[B22] Sun X. Ou Z. Xie M. Kang R. Fan Y. Niu X. (2015). HSPB1 as a novel regulator of ferroptotic cancer cell death. Oncogene 34, 5617–5625. 10.1038/onc.2015.32 25728673 PMC4640181

[B23] Sung H. Ferlay J. Siegel R. L. Laversanne M. Soerjomataram I. Jemal A. (2021). Global cancer statistics 2020: GLOBOCAN estimates of incidence and mortality worldwide for 36 cancers in 185 countries. CA a Cancer J. Clin. 71, 209–249. 10.3322/caac.21660 33538338

[B24] Takano Y. Shida A. Fujisaki M. Mitsumori N. Yanaga K. (2020). Prognostic significance of ZKSCAN3 (ZNF306) expression in gastric carcinoma. Anticancer Res. 40, 81–86. 10.21873/anticanres.13928 31892555

[B25] Wang H. X. Yang Y. Guo H. Hou D. D. Zheng S. Hong Y. X. (2016). HSPB1 deficiency sensitizes melanoma cells to hyperthermia induced cell death. Oncotarget 7, 67449–67462. 10.18632/oncotarget.11894 27626679 PMC5341888

[B26] Yang L. Hamilton S. R. Sood A. Kuwai T. Ellis L. Sanguino A. (2008a). The previously undescribed ZKSCAN3 (ZNF306) is a novel driver of colorectal cancer progression. Cancer Res. 68, 4321–4330. 10.1158/0008-5472.CAN-08-0407 18519692

[B27] Yang L. Zhang L. Wu Q. Boyd D. D. (2008b). Unbiased screening for transcriptional targets of ZKSCAN3 identifies integrin beta 4 and vascular endothelial growth factor as downstream targets. J. Biol. Chem. 283, 35295–35304. 10.1074/jbc.M806965200 18940803 PMC2596387

[B28] Yang L. Wang H. Kornblau S. M. Graber D. A. Zhang N. Matthews J. A. (2011). Evidence of a role for the novel zinc-finger transcription factor ZKSCAN3 in modulating cyclin D2 expression in multiple myeloma. Oncogene 30, 1329–1340. 10.1038/onc.2010.515 21057542 PMC4128235

[B29] Zhang X. Jing Y. Qin Y. Hunsucker S. Meng H. Sui J. (2012). The zinc finger transcription factor ZKSCAN3 promotes prostate cancer cell migration. Int. J. Biochem. Cell Biol. 44, 1166–1173. 10.1016/j.biocel.2012.04.005 22531714 PMC3358443

[B30] Zhang H. Liu T. Zhang Z. Payne S. H. Zhang B. McDermott J. E. (2016). Integrated proteogenomic characterization of human high-grade serous ovarian cancer. Cell 166, 755–765. 10.1016/j.cell.2016.05.069 27372738 PMC4967013

