# Supplementary material for: ZKSCAN3 promotes ovarian cancer cell proliferation by increasing HSPB1 expression
Source: Front Mol Biosci. 2025 Nov 28;12:1623062. doi: 10.3389/fmolb.2025.1623062 (PMC12698437; doi:10.3389/fmolb.2025.1623062)
Supplement: Supplementary file 1 [file Supplementaryfile1.docx]

**Figure S1 related to Figure 1**

(A). ZKSCAN3 was knocked-down in ovarian cancer cells with lentivirus-expressed shRNA. Cell proliferation of control and ZKSCAN3-KD cells over 4 days was measured with cell counting.

(B). ZKSCAN3 was knocked-down in ovarian cancer cells with lentivirus-expressed shRNA. 1000 cells were seeded into each 3.5cm dish. 12 days later, cells were fixed and then stained with crystal violet.

(C). Control or HSPB1-knockdown cells were labelled with BrdU and then subject to immunostaining with anti-BrdU antibody. The left panel shows representative images while the right panels shows statistical result for four independent images. Scale bars denote 10μm.

(D). Control or ZKSCAN3-KD cells were treated with 0.2μM oxaliplatin for three days and cell proliferation was analyzed with MTT.

(E). Control or ZKSCAN3-KD cells were seeded to top charmbers of transwell inserts and subject to transwell assay. Cells migrated through the insert membrane were stained with crystal violet. The left panel shows representative images of migrated cells while the right panels shows the statistic result of three independent images. Scale bars denote 100μm.

(F). Control or ZKSCAN3-KD cells were seeded into 12-well plates. Cell migration was determined by the cell ability to fill in a wound created by scratch. Scale bars denote 100μm.

(G). ZKSCAN3 was knocked-down in ovarian cancer cells with lentivirus-expressed shRNA. A shRNA-resistant ZKSCAN3 cassette was rescue-expressed with lentivirus. Cell proliferation over 4 days was measured with cell counting.

(H). ZKSCAN3 was knocked-down in ovarian cancer cells with lentivirus-expressed shRNA. A shRNA-resistant ZKSCAN3 cassette was rescue-expressed with lentivirus. 1000 cells were seeded into each 3.5cm dish. 12 days later, cells were fixed and then stained with crystal violet.

(I). ZKSCAN3 was stably overexpressed in ovarian cancer cells with lentivirus transduction. Cell proliferation over 5 days was measured with cell counting.

(J). ZKSCAN3 was stably overexpressed in ovarian cancer cells with lentivirus transduction. 1000 cells were seeded into each 3.5cm dish.12 days later, cells were fixed and then stained with crystal violet.

**Figure S2 related to Figure 2**

Shown are real-time RT-PCR analysis of PFKFB4, ALDH1A3 and PREX1 genes that were significantly changed by ZKSCAN3-KD in RNA-Seq.

**Figure S3 related to Figure 3.**

(A). Cells were analyzed with ChIP. Immunoprecipitation was done with ZKSCAN3 antibody with IgG as control. The left panel shows the result of realtime PCR and the right panel shows the agarose gel image for conventional PCR products.

(B). ZKSCAN3 was knocked-down with lentivirus-expressed shRNA. Whole cell lysate (WCL) were then analyzed with Western Blot (WB).

(C). ZKSCAN3 was stably expressed in A2780 ovarian cancer cells with lentivirus transduction followed by antibiotic selection. Whole cell lysate (WCL) were then analyzed with Western Blot (WB).

**Figure S4 related to Figure 4.**

(A). HSPB1 was knocked-down in ovarian cancer cells with lentivirus-expressed shRNA. Cell proliferation over 4 days was measured with cell counting.

(B). HSPB1 was knocked-down in ovarian cancer cells with lentivirus-expressed shRNA. 1000 cells were seeded into each 3.5cm dish. 12 days later, cells were fixed and then with crystal violet.

(C). Control or HSPB1-knockdown cells were labelled with BrdU and then subject to immunostaining with anti-BrdU antibody. Scale bars denote 10μm.

(D). HSPB1 was overexpressed in ovarian cancer cells. Cell proliferation over 5 days was measured with cell counting.­

(E). HSPB1 was overexpressed in ovarian cancer cells. 1000 cells were seeded into each 3.5cm dish. 12 days later, cells were fixed and then with crystal violet.

(F). HSPB1 was expressed with lentivirus-transduction in ZKSCAN3-KD cells. Cell proliferation over 4 days was then measured with cell counting.

(G). HSPB1 was rescue-expressed with lentivirus in ZKSCAN3-KD cells. 1000 cells were seeded into each 3.5cm dish. 12 days later, cells were fixed and then with crystal violet.
