# Supplementary material for: ZKSCAN3 promotes ovarian cancer cell proliferation by increasing HSPB1 expression
Source: Front Mol Biosci. 2025 Nov 28;12:1623062. doi: 10.3389/fmolb.2025.1623062 (PMC12698437; doi:10.3389/fmolb.2025.1623062)
Supplement: Supplementary file 2 [file Table1.docx]

| **name** | **sequence (5'-->3')** |
| --- | --- |
| HSPB1-ChIP PCR-F | CACAAACCTAGATGGTCTAGC |
| HSPB1-ChIP PCR-R | ACTTACCCTCATGTTGCAGCT |
| PFKFB4-RT-F | CAACATCGTGCAAGTGAAACTG |
| PFKFB4-RT-R | GACTCGTAGGAGTTCTCATAGCA |
| ALDH1A3-RT-F | TGAATGGCACGAATCCAAGAG |
| ALDH1A3-RT-R | CACGTCGGGCTTATCTCCT |
| PREX1-RT-F | GGCATTCCTGCATCGCATC |
| PREX1-RT-R | CGGGTGTAAACAATACTCCAAGG |
| ACTB-RT-F | AGAGCTACGAGCTGCCTGAC |
| ACTB-RT-R | AGCACTGTGTTGGCGTACAG |
| HSPB1-RT-F | ACGGTCAAGACCAAGGATGG |
| HSPB1-RT-R | AGCGTGTATTTCCGCGTGA |
| ZKSCAN3-shRNA1-F | CCGGAGTAGCCAATTTCAGCTAATGTACTAGTCATTAGCTGAAATTGGCTACTTTTTTT |
| ZKSCAN3-shRNA1-R | AATTAAAAAAAGTAGCCAATTTCAGCTAATGACTAGTACATTAGCTGAAATTGGCTACT |
| ZKSCAN3-shRNA2-F | CCGGGCCTCCTTGAACATCACAGAATACTAGTTTCTGTGATGTTCAAGGAGGCTTTTT |
| ZKSCAN3-shRNA2-R | AATTAAAAAGCCTCCTTGAACATCACAGAAACTAGTATTCTGTGATGTTCAAGGAGGC |
| HSPB1-shRNA1-F | CCGGGATCACCATCCCAGTCACCTTCTCGAGAAGGTGACTGGGATGGTGATCTTTTT |
| HSPB1-shRNA2-R | AATTAAAAAGATCACCATCCCAGTCACCTTCTCGAGAAGGTGACTGGGATGGTGATC |
| HSPB1-shRNA2-F | CCGGCCGATGAGACTGCCGCCAAGTCTCGAGACTTGGCGGCAGTCTCATCGGTTTTT |
| HSPB1-shRNA2-R | AATTAAAAACCGATGAGACTGCCGCCAAGTCTCGAGACTTGGCGGCAGTCTCATCGG |

**Table 1. Sequence of oligos**
