# Supplementary figures and images for: ZKSCAN3 promotes ovarian cancer cell proliferation by increasing HSPB1 expression
Source: Front Mol Biosci. 2025 Nov 28;12:1623062. doi: 10.3389/fmolb.2025.1623062 (PMC12698437; doi:10.3389/fmolb.2025.1623062)

**A**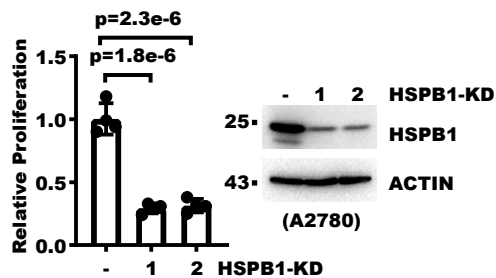**B**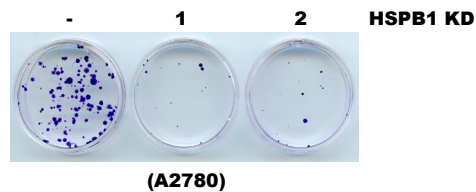**C**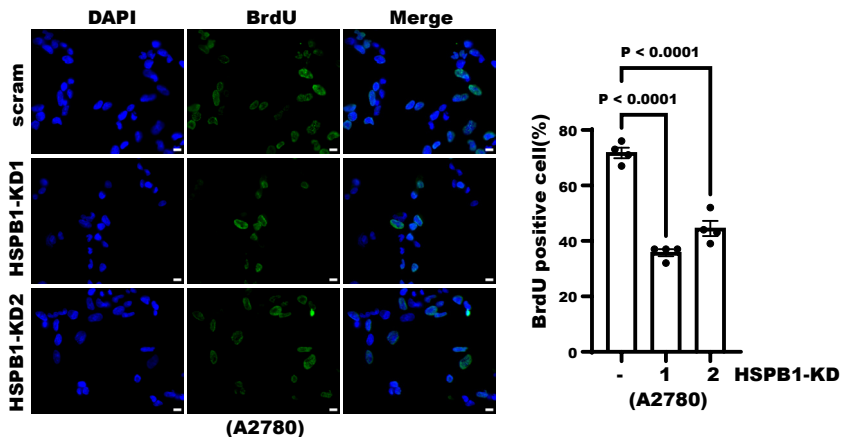**D**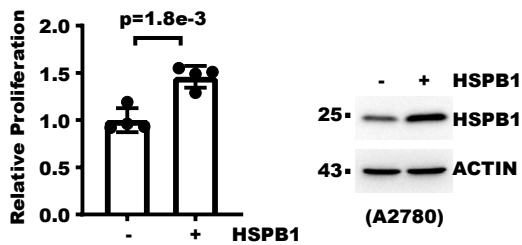**E**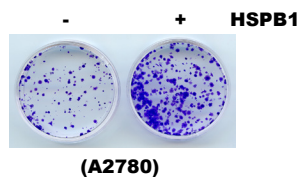**F**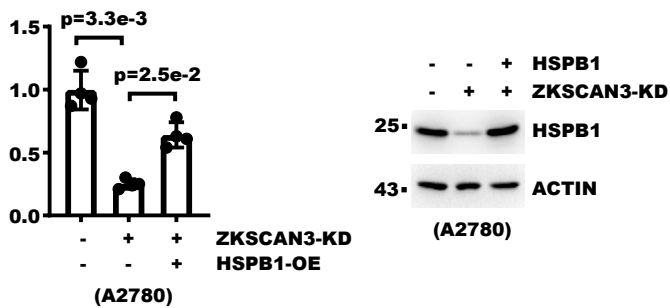**G**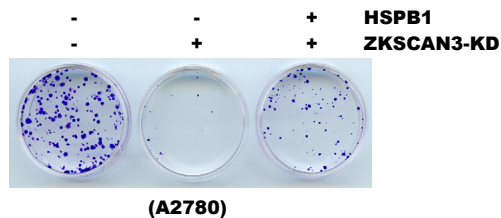

Supplement: Supplementary file 3 [file Image4.pdf]

**A**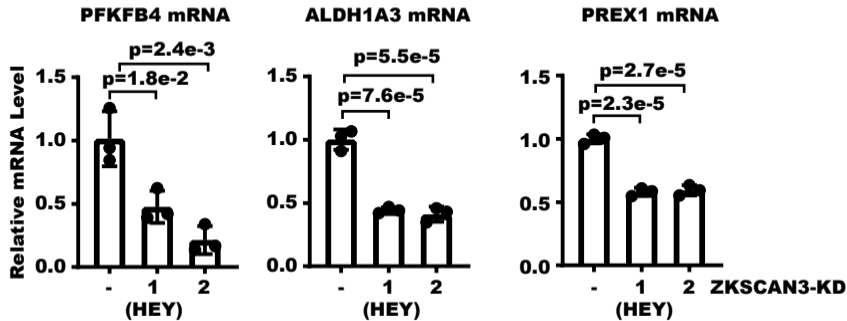

Supplement: Supplementary file 4 [file Image2.pdf]

**A**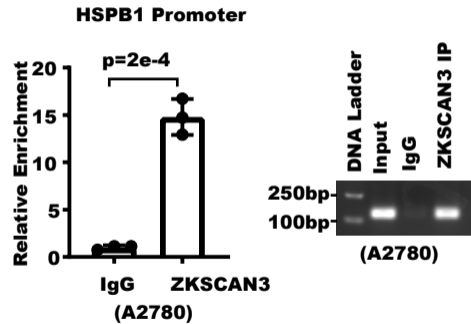**B**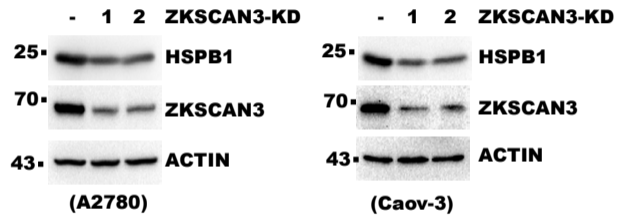**C**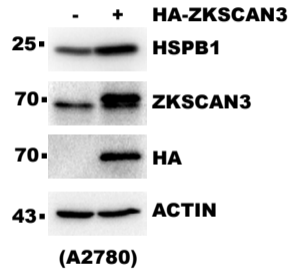

Supplement: Supplementary file 5 [file Image3.pdf]

A

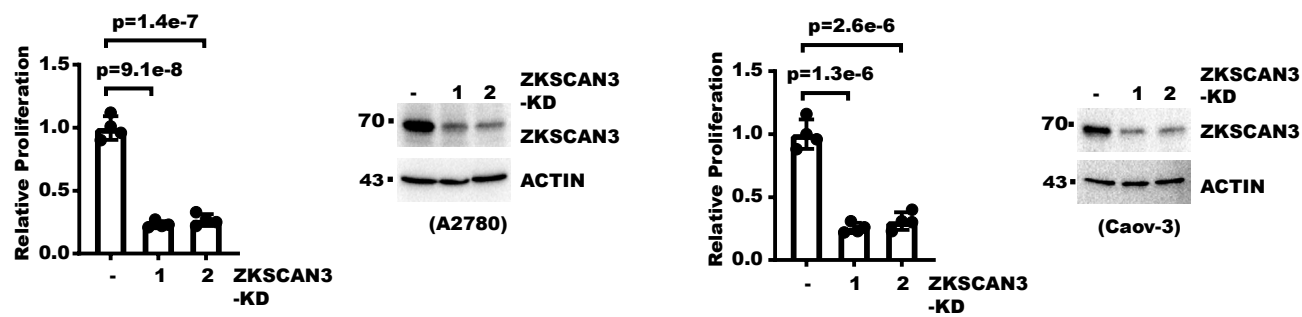

B

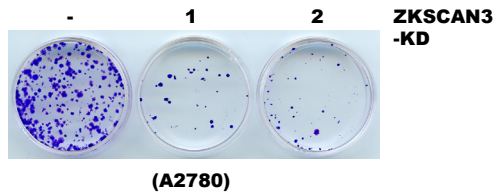

C

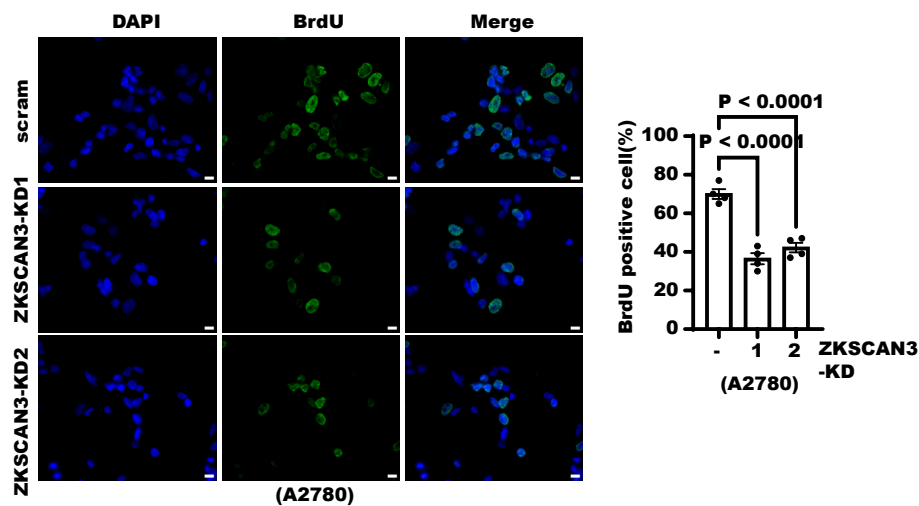

D

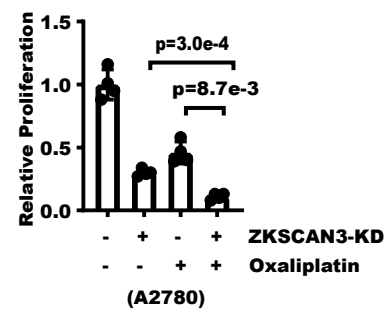

E

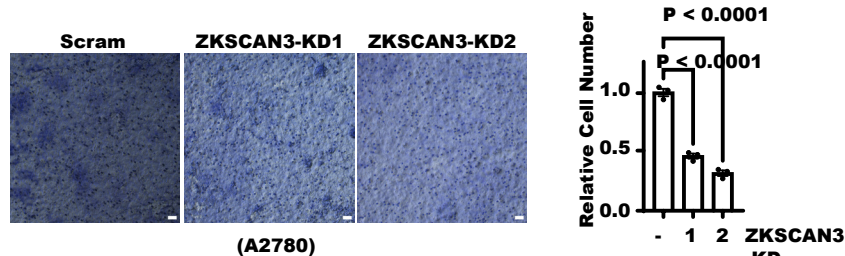

F

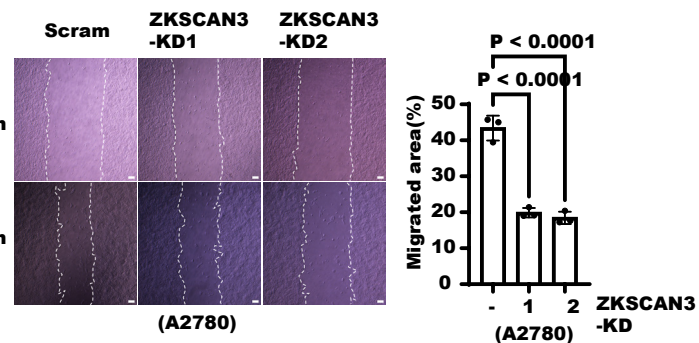

G

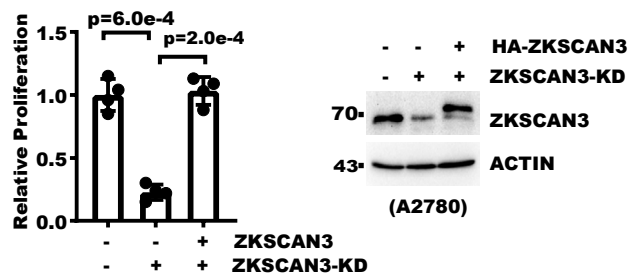

H

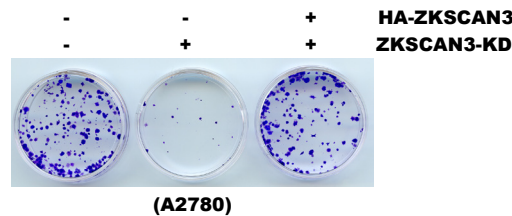

I

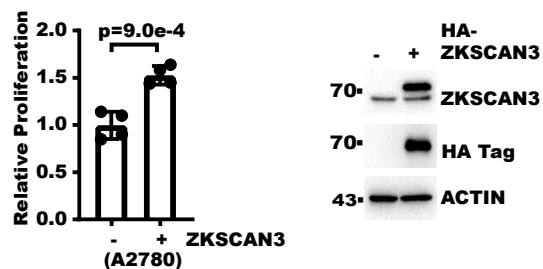

J

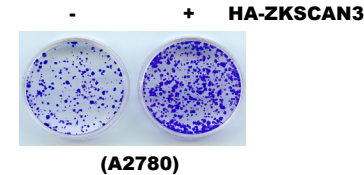

Supplement: Supplementary file 6 [file Image1.pdf]
